# Risodontrypy

**Published:** 1854-04

**Authors:** 


### ARTICLE XV.

### *Risodontrypy.*

This operation, as the drilling of the teeth is now termed, has given rise from time to time to a great deal of contradictory discussion, without either side arriving at any satisfactory conclusions. For our own part, we cannot discover any more favorable features in the *modernized* method of performing the operation than characterized the former. To sustain this position, we will remark that every case of perforating the gum and alveolus which has fallen under our observation, has, in a short time after, assumed the same condition as though it had been drilled under the free margin of the gum at first. The sero-purulent matter and blood escaping from the orifice in the tooth excites absorption of the thin process of the border of the alveolus, and dissects the gum loose from the neck of the tooth, and escapes by that means at last, and the perforation of the gum heals as any other wound. Again, as the wound of the pulp by decay is always a direct and local cause of pain in a tooth, we cannot understand how making an additional wound with a drill, can render the tooth less liable to pain and disease. The advantage proposed to be gained by drilling through the gum, of shutting out the air and irritating fluids of the mouth from the pulp, does not seem to be worth considering, since the free margin of the gum covers the orifice in the tooth as securely as the perforated opening in the gum; and besides, an opening which would give free exit, either through the gum or under it, to the accumulating contents of the pulp cavity, would as well give ingress to air and the fluids of the mouth. As long as the pulp is in a normal condition, a large amount of blood circulates through it, but as soon as it is irritated by any cause, the determination of blood to it increases, and must extravasate where the pulp is wounded, or violent congestion, or engorgement of the pulp vessels and surrounding tissues ensues. Now, it would seem to us, that under such circumstances the nearer the artificial opening is made to the original wound, the better, because the blood and pus which accumulates at the extremity of the pulp cavity, as it is opposite that point that it is most often exposed, must regurgitate upon the pulp until it finds vent through the artificial opening, which regurgitation would soon break up the structure of that substance. This, to us at first view many years ago, induced us to tube the plug, but a short experience soon proved the fallacy of the attempted preservation of the pulp alive. A pulp never remains in a stationary condition; when wounded, suppuration soon sets in, and absorption and sloughing takes place, or fungous granulations are thrown up, which keeps the cavity constantly full, and sometimes become so abundant as to fill the whole cavity of decay; and thus, as long as it is in the tooth, it is subject to change, and needs an external opening. That a tooth will give rise to pain when plugged over the pulp, seems now to be conceded on all sides; and on account, too, of the accumulating fluids in the pulp cavity, this suggests, at once, the operation of drilling an orifice, which shall be less objectionable than the former cavity. It requires no stretch of the imagination to see the propriety of this procedure, as a method of giving temporary relief from suffering; but, if we stop at this point in our efforts to save a valuable organ of mastication and appearance, we deserve less credit for our skill than belongs to us for duties well performed, and that looks into the future for the test of merited reward. This operation, in the majority of cases, is only quieting the patient's anxieties until the destruction of the organ is irretrievably effected.

Again, in perforating below the margin of the gum, a number of membranes, differing very much in character, are wounded; first, the mucous membrane; second, the alveolo-dental membranes; third, the dentine and the internal membrane of the pulp cavity. Now, this makes a very severe wound, so far as the nature of the tissues of the parts are concerned, and to restore it to a reasonable state of health, some claim for the process, that ossification of the pulp takes place outside of the point at which the pulp cavity is perforated, and that the orifice drilled through the tooth also becomes filled up with ossific matter, and in this way the life of the tooth is saved, and the surrounding parts become whole. We see cases of drilled teeth every day, and have as yet seen no traces of such changes having taken place, nor can we at present regard the matter as deserving serious reflection. We very frequently find granules of ossific matter occupying the pulp cavity, enveloped by a thin vascular and sensitive membrane, similar to periosteum, when extirpating the pulp, but never regarded this as resulting from any effort of nature to change the character of the pulp from its sensitive and vascular condition to that of an ossific substance, to ward off the consequences of exposure, to the liability to disease or suffering. We have frequently found such granules on breaking open sound teeth, and in cases where the pulp had become exposed by rapid decay. We recollect sending a specimen of such ossification in the pulp cavity—but not united to it—some years ago, to Prof. C. A. Harris, of Baltimore, the reception of which he acknowledged in the American Journal, at the time. This specimen, as well as many others, was discovered fifteen years ago, while making experiments to *fossilize* the pulp. These anamolies are the results of modified functions in the pulp substance, which we, as yet, have no means of rousing up or controlling. Every effort, therefore, to preserve the pulp alive, or to cause it to assume or take on a modified state of existence to that end, is but experimental. It has been claimed also for this operation, that it is founded on *true surgical principles.* "*Surgery, properly,* is the act of healing by manual operations; or, that branch of medical science which treats of manual operations for the healing of diseases or injuries of the body," and "*a solution of continuity*, is any division of parts previously continuous, as a wound or a fracture," &c. Now, if a tooth's pulp is wounded by a loss of the bone, by decay or other cause, is it surgery to make another wound of greater extent than the former, involving the same tissue, as well as other important ones? This is not treating the *wound* first made by manual operation, but making *another*. Now, where is the surgical principle that is to promote the healing of the second wound? Surgery can only place a wounded part in such a condition as best to favor the operations of nature in her efforts to restore a breach of continuity. If an organ or limb is irrecoverably injured, and we cannot, by directing our manual or surgical operations to the *wound itself*, hope to have it restored, and it is a part that is not of vital importance to the economy, it is our duty to extirpate it entirely, nay, our only remedial means. This is *nature's* surgery, so far as it applies to cutting organs loose from the body, when she cannot bring sufficient vital force to operate in the restoration of the wounded member. Does not this apply to the exposed or injured dental pulp, in the mass of cases, if not all, sooner or later, whether plugged or drilled or left exposed without a plug?

This modern method of operating was marshalled before the profession with a strong advance and rear guard, as the ultimatum of scientific treatment of the dental pulp, only lacking for its perfection a more extended experience, and care in its application. With due deference to all who are engaged in its practice, we would assert, that so far as we have been able to observe the results of the method, it is the most pernicious that has yet been devised. There are very few cases in which it will well apply, as a *durable* operation for preserving a tooth, even if it had strong claims in favor of placing a tooth in a comfortable condition. It cannot be applied in deciduous teeth, on account of the pain it excites in its performance. It cannot be applied with any certainty with reference to impinging upon the most desirable point of the pulp in a molar tooth. Within a few days we have removed a plug from a superior first permanent tooth, after a month's suffering, which at last became intolerable, and found that the buccal nerves were dead, and the palatine living and in a high state of inflammation. This would have required to have been drilled on both sides; and, within two months, a patient applied in great suffering from a first superior bicuspis, which had been drilled by one of its most eminent supporters, and upon removing the plug, it was found that the drill had entered the pulp cavity a little way above the neck of the tooth, and had passed out obliquely upwards and backwards into the alveolus. This tooth had been subject to a much longer period of suffering than is usual when alveolar abscess sets in. An instrument could be passed into the posterior opening under the gum, as well as under the gum and into the orifice on the buccal side of the tooth; notwithstanding this *thorough* drilling, there was an alveolar abscess at the extremity of the root. We do not cite this operation as one to condemn the method, when carefully applied, but to show what may happen in the hands of experienced operators. When all these cavities were well plugged, the irritation of the surrounding parts subsided, as well as the fistulous opening from the alveolar abscess in the gum. There were many teeth in the same mouth that had been plugged over the pulp cavities—some before destroying the pulp, and some after—and they had not given rise to as much suffering altogether, as this single case. It is useless to drill a tooth where there is already an unhealthy condition of the pulp, and this is seldom met with when preparing a tooth for plugging, because patients rarely apply to us until they are compelled to do so from suffering, and if a portion of the pulp has been destroyed or become fungoid, drilling will never restore it to a normal condition, and in such a case it is depending upon the same principle for its success, as cases under the former method of drilling under the free margin of the gum. It is not durable, because when the pulp of a tooth becomes dead, the walls of the pulp cavity commence to soften, and in time the whole crown of the tooth, especially if it be a drilled one, discolors, and decay is the inevitable consequence. The only application that we can see for it is, where the pulps of teeth, with incompletely formed roots, are exposed, if drilling would preserve the pulp in a reasonable condition of health until the growth of the roots was completed, and the plug preserve the crown, we can understand how it could be rendered of great service in some cases, but we do not know of that as yet having been urged in its favor. It is not a safeguard against alveolar abscess, as nearly, if not quite all the cases we have seen, where the pulps had become dead, alveolar abscess had set in; we see abscess of course, every day, where teeth are as open as drilling could make them.

It seems very strange that such great difference of opinion and experience should exist between respectable practitioners, about the same mode of operating, where each are equally actuated to do the best that theory and practice could suggest for the good of their patients. It is doubtless true, that some practitioners are more easily satisfied than others, with regard to what constitutes success in treating teeth, when the pulps are exposed. The only way that such difference could be settled, would be by the relative success of a given number of operations under similar circumstances. As far as our experience goes, we have yet to see the first patient who would be willing to have the operation of drilling at all repeated, after they have had a pulp destroyed, removed and the root plugged. This opinion is corroborated by a number of my fellow practitioners, who have had considerable experience in the profession, as well as in this operation, after the different modes that have been suggested. The following case has some point in it:

Miss ———, aged 12 years, presented a case of drilling through the gum and alveolus of a lateral incisor, which had given rise to frequent attacks of pain, and at all times felt uncomfortable. There was a small spongy tumor on the gum opposite the drilled orifice, which had assumed a sensitive character, and prevented the use of the brush over that part of the gum, and was subject to slight bleedings. The tooth presented some discoloration and looseness in the socket. We refused to attempt its treatment, until she had seen and consulted her dentist, partly because he might wish to modify the treatment, and partly because we did not think it fair to risk the successful treatment of a case by plugging the root, where so much had been done to render the case difficult. She consulted her dentist, who at once pronounced it a failure, and that the tooth must be extracted. This, however, was not concurred in, when she returned and requested us to do what we pleased to the tooth, but not to extract it. The plug was removed, and a very small opening found to lead to the pulp cavity; the pulp had sloughed away as far down the root as to the drilled orifice; some bleeding was excited on touching the living remnant of the pulp. The tooth was left in this condition for one day, when the remaining portion of the pulp was destroyed, as far down the root as a small probe could be passed to remove it. A small pledget of cotton was forced under the free margin of the gum and into the drilled opening, as the gum was detached in this direction, and allowed to remain for one day, when it was removed, and another larger introduced and left one day also. When it was removed the orifice could be distinctly seen, and as the gum remained elevated and arched over the orifice as high as the pledget of cotton raised it, this opportunity was taken to fill the orifice firmly with gold, first enlarging the mouth of the opening with a burhead drill, so that a larger plug could be entered than would push through; in this way the opening could be plugged firmly. The gum was now allowed to fall over the plug, as it did over the orifice. In a few days the root was plugged, and no irritation setting in, the external cavity was plugged in a few days after. This case was treated during January, 1853, and has been seen frequently since that time, and as yet presents no unhealthy appearance of the gum. The spongy tumor soon left, and the tooth regained a normal firmness in the socket; the appearance of the gum is healthy, and the body of the tooth less dark than before it was plugged in the root.

This case is but a type of many others of similar character from the same operator, as well as many other cases from operators of less repute for sound judgment and skill. Cases of drilling in the old way, prevailed like an epidemic twelve or fifteen years ago, but become less frequent from year to year, and we hoped would entirely disappear, until recently, when it seems to have gained increased energy under a new form, and notwithstanding the success attending the originators of the change, it is producing a great amount of suffering in bungling hands. J. D. W.

